# Supplementary material for: Supply chain concentration and enterprise financialization: Evidence from listed companies in China’s manufacturing industry
Source: PLoS One. 2023 May 10;18(5):e0285308. doi: 10.1371/journal.pone.0285308 (PMC10171682; doi:10.1371/journal.pone.0285308)
Supplement: Attachment — Submitted filename: Response to Reviewers(Submit).docx [file pone.0285308.s002.docx]

**Response to Reviewers**

First of all, we would like to thank reviewers for your insightful, constructive and helpful comments on our manuscript entitled “**Supply Chain Concentration and Enterprise Financialization: Evidence from Listed Companies in China's Manufacturing Industry**”. We have carefully considered and addressed all the comments and made necessary revisions in the revised manuscript. We provide a point-by-point response to the reviewers’ comments below.

The points raised by the reviewers are written in **bold font,** whereas our responses are shown in normal font, and the quotation of the revised manuscript is shown in *italic font*.

# ACADEMIC EDITOR:

**1. Both reviewers raised questions about the introduction and contribution. Please take them seriously.**

**2. The model obviously has endogenous problems. It is suggested to add endogenous discussion in the robustness test.**

**3.There are a lot of grammatical errors. Should read the manuscript carefully to eliminate those errors and, if possible, have a native speaker to proofread the paper.**

***Response:***

Thank you for your suggestions and reminders. We also thank the reviewers for their comments and suggestions. We will carefully revise the paper verbatim according to the comments and suggestions of the reviewers, in order to meet the requirements of publication.

# Reviewer #1:

**I enjoyed reading your paper and this topic sounds interesting and can be potential of publication. Both major and minor issues for improvement are provided. Before resubmitting, please consider the following:**

***Response:***

Thank you very much for your recognition of the topic of this paper. We will continue to revise carefully according to your suggestions.

1. **How did the findings advance the existing/ previous theory (ies)? The authors need to add contributions to theory and literature explaining how this research fills or advances the existing theories.**
2. **In the introduction, the theoretical contribution should be clearly indicated. Please explain to which area of knowledge you are contributing. It is not clear yet! The manuscript, in my opinion, also has some practical implications that should also be pointed out there.**

***Response:***

Thank you very much for your valuable comments and suggestions. The above two questions are both about the marginal contribution of the article, so they are answered together. In the original manuscript, our marginal contribution writing is relatively vague, leading to less prominent theoretical contributions. In the revised manuscript, we strengthened the writing of this part: First of all, we focused on the research object, put suppliers and customers in the same framework, and filled in the deficiencies of the existing literature; Secondly, the mechanism analysis literature, which analyzes the intermediate mechanism between supply chain concentration and enterprise finance from the perspective of commercial credit, has enriched the corresponding theoretical achievements; Thirdly, the interpretation of the real world has practical significance. The revised part is as follows:

…

*This paper's knowledge fields mainly include supply chain management and enterprise financial investment, so this paper empirically analyzes the impact of supply chain concentration on enterprise financialization. It is noteworthy that many scholars have studied the economic consequences of customer concentration, but few have extended to suppliers and supply chains. In addition, although many scholars have studied customer and enterprise financialization separately, few scholars have studied them together. And there are few studies on the specific impact mechanism of supply chain concentration. Compared with the existing research, this paper deepens the existing research from the internal characteristics of the enterprise to the external environment, promoting the research on customer concentration from the existing overall average effect to a more detailed level.* *There may be marginal contributions from the following aspects: First, Unlike Ak & Patatoukas (2016) and Cao et al. (2021), which only focuses on the impact of customer concentration on enterprise financialization, this paper integrates customer concentration and supplier concentration into the same analytical framework to investigate the impact on enterprise financialization and enrich relevant research on customer concentration. Second, the existing research on the mechanism of supply chain concentration affecting enterprise financialization mainly focuses on the enterprise's own behavior, just like the research of Huang et al. (2016). This paper introduces non-contractual upstream and downstream commercial credit as intermediary variables, and studies the specific impact mechanism which is of great significance for enriching the research on the mechanism of supply chain concentration on enterprise economic behavior. Third, from the perspective of supply chain concentration and enterprise financialization, this paper provides new evidence for mandatory disclosure of customer information from both risk and income aspects, and then provides reference for subsequent policy formulation.*

…

3. **It is worth referring to the following studies:**

**Jum’a, L., Zimon, D., & Ikram, M. (2021). A relationship between supply chain practices, environmental sustainability and financial performance: evidence from manufacturing companies in Jordan. Sustainability, 13(4), 2152.**

**Gupta, S., & Dutta, K. (2011). Modeling of financial supply chain. European journal of operational research, 211(1), 47-56. etc.**

***Response:***

Thank you for your recommendations. I have carefully read the content and framework of these two articles and grasped the essence and basic content of supply chain management. In addition, I have applied these two articles three times in this paper to draw out the research object. First of all, citing Gupta & Dutta (2011) on the essence of supply chain management, it is explained that supply chain management revolves around the coordination and cooperation among several business partners linked by material, capital and information flows. Then the article of Jum’a et al. (2021) is cited to illustrate the importance of supply chain relationship in supply chain management. Then, the article of Gupta & Dutta (2011) is quoted again to explain the types of supply chain partners and the main research objects of this article. The revised part is as follows:

…

*Supply chain management focuses on coordination and cooperation among several business partners linked by material, capital and information flows (Gupta & Dutta, 2011). The practice of supply chain management includes four parts: supply chain relationship, information sharing level, information quality level and delay (Jum’a et al., 2021). Among them, customer relationship is the most basic content. Supply chain partners include suppliers of basic raw materials and parts, manufacturers, wholesalers, distributors, transporters, retailers, banks and financial institutions (Gupta & Dutta, 2011). In this paper, it is generally summarized as upstream suppliers and downstream customers.*

…

4. **Unfortunately, the** **limitations of the study were not included in the Conclusions section, though they should be because they certainly exist.**

***Response:***

Thank you very much for your valuable comments and suggestions. The manuscript does not point out the limitations of the paper. There are the following limitations in the scope of application and endogenous test of this paper. The revised part is as follows:

.…

*There are also some limitations in this paper: First, this paper only studies the samples of listed companies, but the research of non-listed companies is not involved in this paper. So, the conclusion of this paper is only applicable to listed companies. Second, there are still some problems in the endogenous treatment of this paper. Compared with the enterprise financialization, the customer concentration is not an exogenous variable, so it is difficult to find an instrumental variable that does not affect the enterprise financialization at all.*

…

5. **It does not demonstrate its innovation and contribution with the comparison of the existing studies, which is a most important key for possible publication.**

***Response:***

Thank you very much for your valuable comments and suggestions. The manuscript does not prove its innovation and contribution through comparison with existing research. After studying relevant research, this paper believes that the defects of the existing literature are as follows. Many scholars have studied the economic consequences of customer concentration, but few have extended to suppliers and supply chains. And few scholars have studied the specific impact mechanism of supply chain concentration. Therefore, compared with the existing research, the revised draft has made innovations and contributions in research content, research mechanism and research. This paper deepens the existing research from the internal characteristics of the enterprise to the external environment, promoting the research on customer concentration from the existing overall average effect to a more detailed level. The revised part is as follows:

.…

*This paper's knowledge fields mainly include supply chain management and enterprise financial investment, so this paper empirically analyzes the impact of supply chain concentration on enterprise financialization. It is noteworthy that many scholars have studied the economic consequences of customer concentration, but few have extended to suppliers and supply chains. In addition, although many scholars have studied customer and enterprise financialization separately, few scholars have studied them together. And there are few studies on the specific impact mechanism of supply chain concentration. Compared with the existing research, this paper deepens the existing research from the internal characteristics of the enterprise to the external environment, promoting the research on customer concentration from the existing overall average effect to a more detailed level. There may be marginal contributions from the following aspects: First, Unlike Ak & Patatoukas (2016) and Cao et al. (2021), which only focuses on the impact of customer concentration on enterprise financialization, this paper integrates customer concentration and supplier concentration into the same analytical framework to investigate the impact on enterprise financialization and enrich relevant research on customer concentration. Second, the existing research on the mechanism of supply chain concentration affecting enterprise financialization mainly focuses on the enterprise's own behavior, just like the research of Huang et al. (2016). This paper introduces non-contractual upstream and downstream commercial credit as intermediary variables, and studies the specific impact mechanism which is of great significance for enriching the research on the mechanism of supply chain concentration on enterprise economic behavior. Third, from the perspective of supply chain concentration and enterprise financialization, this paper provides new evidence for mandatory disclosure of customer information from both risk and income aspects, and then provides reference for subsequent policy formulation.*

…

**Thank you again for your comments and suggestions. Your review opinions are the driving force for our progress and the direction of our efforts. We hope you will review our paper again and look forward to your further comments.**

# Reviewer #2:

**This paper selects the panel data of A-share listed companies in China from 2009 to 2021, uses customer concentration and supplier concentration to measure supply chain concentration, and examines the impact of supply chain concentration on enterprise financialization. The results show that both customer concentration and supplier concentration can significantly promote the financialization of enterprises, while inhibiting the substantiation of enterprises. The conclusion is still valid after robustness and endogenous treatment; The analysis of influence mechanism shows that customer concentration affects enterprise financialization by increasing business credit, while supplier concentration has no similar intermediary effect; The results of heterogeneity analysis show that the impact of supply chain concentration on enterprise financialization is significantly different in state-owned enterprises and small and medium-sized enterprises, and more significant in enterprises with less fierce industry competition and located in the central and western regions. The research in this paper provides empirical evidence for restraining the adverse impact of enterprise financialization on industry and taking advantage of the benefit effect brought by supply chain concentration.**

***Response:***

Thank you for your attention to the paper. We will carefully consider and make comprehensive modifications according to your suggestions and opinions, so as to meet the requirements of journal publication.

1. **The aim of the analysis should be evidenced in the abstract and introduction sections. The methodology should be further explained.**

***Response:***

Thank you very much for your valuable comments and suggestions. First of all, we have made a comprehensive revision to the abstract, reflecting the background of the article, research purpose, research data, research methods and research results. The revised part is as follows:

…

*Enterprise financialization will block the equipment update and technological innovation of enterprises by crowding out the main business funds. The risks and benefits of supply chain concentration will affect the enterprise financialization. This paper selects the panel data of A-share listed companies in China from 2009 to 2021, and uses fixed effect regression to analyze the impact of supply chain concentration on enterprise financialization. The conclusions show: both suppliers and customers concentration significantly promote the financialization of enterprises, and this conclusion is still valid after a series of tests; This kind of financialization effect is heterogeneous in four aspects: the nature of property rights, the scale of enterprises, the intensity of industrial competition and the level of economic development in the region where the enterprises are located; the mechanism analysis show that customer concentration can affect enterprise financialization through upstream commercial credit, but supplier concentration cannot affect enterprise financialization through downstream commercial credit.*

…

Secondly, we have made four revisions to the introduction. First of all, it points out the consequences of enterprise financialization and the current situation of enterprise financialization in China. Then introduce the essence of supply chain management and the current situation of supply chain concentration in China. Next, it leads to the purpose of this paper, and analyzes the possible impact of supply chain concentration on enterprise financialization from two aspects. Finally, it points out the contribution and innovation of this paper compared with the existing researches. The revised part is as follows:

.…

*Enterprise financialization will block the equipment update and technological innovation of enterprises by crowding out the main business funds. The risks and benefits of supply chain concentration will affect the enterprise financialization. Therefore, the main purpose of this paper is to study the impact of supply chain concentration on enterprise financialization. Based on this, this paper selects the relevant panel data of 17600 A-share listed companies in China from 2009 to 2021, and uses double fixed effect regression to analyze the hypothesis. In order to deepen the research, this paper also analyzes the intermediary role of commercial credit between supply chain concentration and enterprise financialization. In addition, this paper also analyzes the heterogeneity from four aspects: the nature of property rights, the size of enterprises, the intensity of industrial competition and the level of economic development in the region where the enterprises are located This research provides guidelines for policy makers, helps managers improve supply chain relations and offers empirical evidence to curb the adverse impact of corporate financialization.*

…

2. **The results should be discussed in terms of policy implications.**

***Response:***

Thank you very much for your valuable comments and suggestions. The policy recommendations in the manuscript were not analyzed item by item based on the research conclusions. The revised manuscript puts forward corresponding policy recommendations for the conclusions of benchmark regression, mechanism analysis and heterogeneity analysis. The revised part is as follows:

…

*Based on the above research results, this paper puts forward the following policy recommendations: First, as the concentration of supply chain aggravates the financialization of enterprises, the government needs regulate the profit-seeking behavior and promote the construction of positive cooperative relationship between upstream and downstream enterprises in the supply chain. With regard to whether the CSRC has required the disclosure of supply chain information, this paper believes that regulators should not only disclose the sales information of the top five customers, but also require further disclosure of the basic characteristics of upstream and downstream enterprises, so as to help information users more objectively evaluate the business status of enterprises. Moreover, according to the analysis of commercial credit as an intermediary variable mechanism, the government should pay attention to the important role of enterprise commercial credit as an informal financing channel, improve the relevant laws and regulations on debt governance, and protect the interests of creditors from both legal and moral aspects. Finally, according to the results of the heterogeneity analysis, the government should actively provide a good business environment for enterprises, give certain policy support to enterprises with fierce industrial competition and enterprises in the midwestern regions, and support the development of minor enterprises.*

…

3. **The conclusions should be improved with the weaknesses of the analysis and the insights for future research.**

***Response:***

Thank you very much for your valuable comments and suggestions. This paper has some limitations in the research scope and endogenous test, and puts forward suggestions for future research. The revised part is as follows:

…

*There are also some limitations in this paper: First, this paper only studies the samples of listed companies, but the research of non-listed companies is not involved in this paper. So, the conclusion of this paper is only applicable to listed companies. Future research can be expanded according to the unlisted companies. Second, there are still some problems in the endogenous treatment of this paper. Compared with the enterprise financialization, the customer concentration is not an exogenous variable, so it is difficult to find an instrumental variable that does not affect the enterprise financialization at all. Future research will try to find out the policy impact that affects the concentration of corporate customers, and carry out research with the method of policy identification.*

…

4. **Finally, the manuscript should be English proofread because some sentences are not clear.**

***Response:***

Thank you very much for your valuable comments and suggestions. The manuscript has been polished by scholars majoring in English, which the sentences and paragraphs that are incorrect in grammar, inappropriate in wording and not in line with the rigor of the paper have been revised.

**Thank you again for your comments and suggestions. Your review opinions are the driving force for our progress and the direction of our efforts. We hope you will review our paper again and look forward to your further comments.**
